# Challenges in Evaluating Pediatric Fever and Rash in the Era of COVID-19 and Multisystem Inflammatory Syndrome in Children (MIS-C)

**DOI:** 10.7759/cureus.21764

**Published:** 2022-01-31

**Authors:** Taylor Jarvill, Priyanka Lauber, Samuel Umaru, Tibisay Villalobos, Susan K Yaeger

**Affiliations:** 1 Department of Emergency and Hospital Medicine, University of South Florida Morsani College of Medicine, Lehigh Valley Health Network Campus, Allentown, USA; 2 Department of Pediatrics, University of South Florida Morsani College of Medicine, Lehigh Valley Health Network Campus, Allentown, USA

**Keywords:** pediatric fever, fluid-refractory shock, pediatric covid-19, case report series, multisystem inflammatory syndrome in children

## Abstract

Introduction: Severe acute respiratory syndrome coronavirus 2 (SARS-CoV-2) has challenged the medical community to characterize and treat a new illness. Now almost two years after the initial confirmed cases of COVID-19, medical teams are faced with another unique disease process temporally related to the pandemic-multisystem inflammatory syndrome in children (MIS-C). The comparison of these patients’ presentations illustrates the new challenges of evaluating a pediatric rash and fever in the era of MIS-C.

Case Reports: This report presents three cases with features of MIS-C, positivity for SARS-CoV-2, rashes, fevers, gastrointestinal involvement, and elevated inflammatory markers. The first case poses a diagnostic dilemma. While the case 1 patient has many features of MIS-C, his nasal swab was positive for Methicillin-sensitive *Staphylococcus Aureus* (MSSA). While the second case falls into the case definition of MIS-C, the case 2 patient also met the criteria for atypical Kawasaki disease. Although the third case was positive for SARS-CoV-2, the patient comparatively had a mild elevation of inflammatory markers and a stable clinical course led the treatment team to be more suspicious of immunoglobulin A (IgA) vasculitis versus hand, foot, and mouth disease. The variability in skin rash in patients with MIS-C contributes to the challenge of correctly diagnosing and managing pediatric patients with fever and rash in the emergency department (ED).

Conclusion: Although most children infected with SARS-CoV-2 are asymptomatic or present with mild respiratory illness, it is critical to recognize children at-risk for fluid-refractory shock in MIS-C. With the continuing SARS-CoV-2 pandemic, emergency department (ED) providers will have to be alert and have high suspicion when evaluating a child with a fever and a rash to properly identify children presenting with this serious illness.

## Introduction

Fever and rash are common presentations in the pediatric emergency department (ED). According to the 2017 National Hospital Ambulatory Medical Care Survey, fever was the most common principal reason for an ED visit for children under age 15 [1]. Skin rash was the third and sixth most common chief complaint made by females and males, respectively, in the ED [1]. The differential diagnosis of fever and rash can range from benign to life-threatening conditions and can represent possible public health concerns [2]. With the onset of the severe acute respiratory syndrome coronavirus 2 (SARS-CoV-2) pandemic, a new condition known as a multisystem inflammatory syndrome in children (MIS-C) emerged, further confounding establishing the correct diagnosis for a child with a fever and rash. A case of MIS-C is defined by the Centers for Disease Control and Prevention (CDC) as an individual aged less than 21 years with a fever over 24 hours, laboratory evidence of inflammation, clinically severe illness requiring hospitalization, evidence of greater than two organ systems involved, a recent SARS-CoV-2 infection or exposure, and no alternative plausible cause [3]. The first identified cases were characterized by fever, rash, gastrointestinal symptoms, elevated inflammatory markers, anemia, and lymphopenia, with concerns for overlapping features with Kawasaki disease (KD) and toxic shock syndrome (TSS) [3-5]. Correctly differentiating MIS-C from other febrile rashes is important in initiating treatment and proper clinical monitoring. A review found that for patients with MIS-C, 80% require intensive care, 48% require vasoactive support, and 20% require mechanical ventilation [6]. This report presents three children: aged two, eight, and 12, who presented to the ED with a rash fever and were positive for SARS-CoV-2. For all three, MIS-C was considered on the differential; however, their presentations were also consistent with other illnesses. The comparison of these patients’ presentations illustrates the new challenges of evaluating a pediatric rash and fever in the era of MIS-C. 

## Case presentation

Case 1

**Figure 1:**
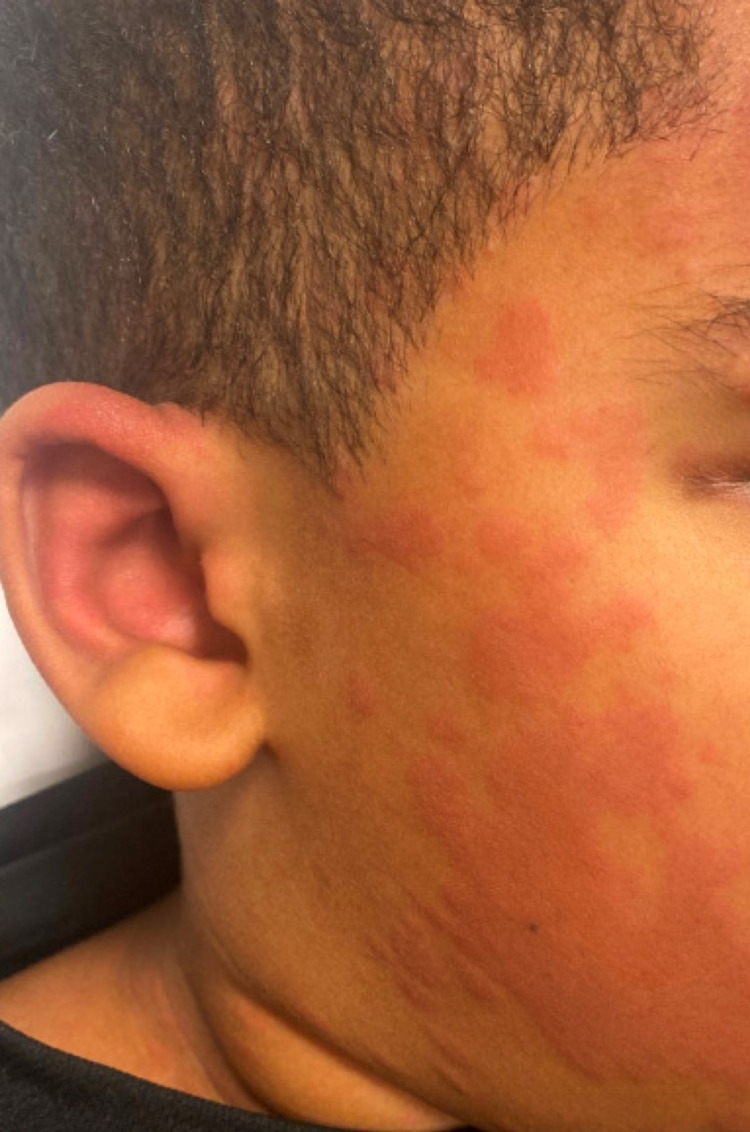
Raised blanching erythematous, non-pruiritic rash.

An obese 12-year-old male with a history of elevated hemoglobin A1C (HbA1c) presented to the ED with five days of fever up to 104⁰ Fahrenheit. Two days before his admission, he tested positive for SARS-CoV-2 by polymerase chain reaction, and before his illness, several family members had also been ill with the virus. He had several days of myalgias, fatigue, generalized abdominal pain, vomiting/diarrhea, and diffuse headache. On examination, he was ill-appearing with fever, tachypnea, tachycardia, and hypotension. A raised blanching erythematous, non-pruritic rash diffusely covered his face, trunk, and extremities (Figure 1). His eyes were injected, as he had subconjunctival hemorrhages. His lips were red and swollen. Laboratory findings at admission were significant for anemia, lymphopenia, hypoalbuminemia (2.5 g/dL) (normal range 3.5-4.7 g/dL) as well as elevations in D-dimer (4.29 ug/mL fibrinogen-equivalent units (FEU)) (normal range <0.50 ug/mL FEU), procalcitonin (30.5 ng/mL) (procalcitonin> or equal to 10 ng/mL is important systemic inflammatory response, almost exclusively due to severe bacterial sepsis or septic shock), C-reactive protein (210 mg/L) (normal range <7.0 mg/L), ferritin (649 ng/mL) (normal range 22-322 ng/mL), and troponin (0.19 ng/mL) (normal range <0.05 ng/mL). The initial chest radiograph appeared normal. His hypotension was refractory to fluid resuscitation with 60 ml/kg of normal saline. He was treated with broad-spectrum antibiotics and initially placed on a high-flow nasal cannula to improve his breathing. He required inotropic support, albumin infusions, and non-invasive positive pressure ventilation in the pediatric intensive care unit (PICU). He was enrolled in a compassionate use trial of remdesivir. Two echocardiograms, performed on day two and day four of hospital stay, showed no myocardial depression or coronary artery abnormalities. Repeat chest radiograph demonstrated diffuse effusions likely secondary to capillary leak. His antibiotics were tailored to cefazolin when cultures were reported sterile and nasal screen was positive for Methicillin-sensitive Staphylococcus Aureus (MSSA). After seven days of critical care, he recovered for three days on the pediatric floor and then was discharged to his also recovered family members with pediatric cardiology and pediatric infectious disease follow-up.

Case 2

A two-year-old male with no relevant past medical history presented to the ED with five days of fever up to 103⁰ F. The father was positive for SARS-CoV-2 in the previous month, and the patient was positive for the immunoglobulin G (IgG) antibody. The family reported increased fussiness, rhinorrhea, emesis, decreased oral intake, decreased urine output, and decreased bowel movements. On exam, an erythematous papular rash was noted on both cheeks, back, and stomach. Cracked, red lips and bilateral conjunctival injection were noted. The patient overall appeared tired with persistent fever and tachycardia. Laboratory findings at admission were significant for lymphopenia, thrombocytopenia (platelets 125 thou/cmm) (normal range 202-403 thou/cmm), hyponatremia (sodium 133 mmol/L) (normal range 135-145 mmol/L), elevated D-dimer (5.77 ug/mL FEU) (normal range <0.50 ug/mL FEU), elevated procalcitonin (8.2 ng/mL) (procalcitonin > 2ng/mL sepsis is likely, unless other causes are known), elevated C-reactive protein (223 mg/L) (normal range <7.0 mg/L), elevated ferritin (474 ng/mL) (normal range 22-322 ng/mL), elevated erythrocyte sedimentation rate (ESR) (58 mm/hr) (normal range 3-13 mm/hr), elevated brain natriuretic peptide (BNP) (3536 pg/mL) (normal range <125 pg/mL), hypoalbuminemia (2.8 g/dL) (normal range 3.5- 4.5 g/dL), and mildly elevated transaminases (AST 70 U/L and ALT 66 U/L) (normal range 26-55 U/L and <56 U/L). Labs taken on day two were significant for anemia (hemoglobin 10.1 g/dL) (normal range 10.2- 12.7 g/dL), and day five showed elevated IL-6 (73 pg/mL) (normal range <5=pg/mL). Chest radiograph from ED visit the previous day demonstrated mild peribronchial interstitial thickening. Three echocardiograms, performed on days one, three, and six of hospital stay, showed no myocardial depression or coronary artery abnormalities. The patient was initially admitted to the pediatric floor and overnight had multiple episodes of emesis. He was started on intravenous immunoglobulin (IVIG) to treat both atypical Kawasaki disease and MIS-C. He then deteriorated, becoming edematous with decreased urine output and requiring multiple fluid boluses for hypotension. He required further inotropic support, albumin infusions, and non-invasive positive pressure ventilation in the PICU. Repeat chest radiograph showed diffuse pleural effusions likely secondary to capillary leak. He was maintained on broad-spectrum antibiotics for initial concerns for septic shock; however, his blood cultures remained negative. After six days of critical care, he was discharged home on 5 mg/kg aspirin per treatment guidelines for atypical Kawasaki disease and MIS-C, with plans to follow up with pediatric cardiology and pediatric infectious disease.

Case 3

An eight-year-old previously healthy female presented to the ED with one day of fever to 103.3⁰ Fahrenheit and several days of a diffuse erythematous, pruritic rash. The patient had no known COVID-19 exposures, was negative by PCR, but was positive for the IgG antibody. She had two days of associated body aches, fatigue, abdominal pain, headaches, and left ankle pain. There was no diarrhea, nausea, shortness of breath, sore throat, vomiting, or wheezing. On exam, she was ill-appearing but alert, active, and vigorous. She was febrile, non-tachycardic, and normotensive. Mild dry mucous membranes with erythema and swelling of the lower lip were noted with left-sided lymphadenopathy. Skin exam showed a diffuse papular, non-blanching, erythematous rash on palms, soles of feet, torso, and bilateral lower extremities. There was swelling and pain with the rotation of the left ankle. The abdomen was tender to deep palpation over the left side and periumbilical area. Laboratory findings at admission were significant for elevated D-dimer (1.69 ug/mL FEU) (normal range <0.50 ug/mL FEU), elevated ESR (34 mm/hr) (normal range 3-13 mm/hr), elevated C-reactive protein (20.8 mg/L) (normal range <7.0 mg/L), hypoalbuminemia (2.9 g/dL) (normal range 3.5- 4.7 g/dL), elevated lactate dehydrogenase (LDH) (286 U/L) (normal range 157-272 U/L), and elevated IL-6 (2.4 pg/mL) (normal range <=5 pg/mL). Complete blood count (CBC), ferritin, and BNP were unremarkable. The patient was admitted to the pediatric floor and received supportive care with intravenous (IV) fluids and nonsteroidal anti-inflammatory drugs (NSAIDS). She defervesced within one day of admission, and after 24 hours afebrile with improvement in her rash, she was discharged with close follow-up with her phencyclidine (PCP).

## Discussion

All three cases had features of MIS-C, with positivity for SARS-CoV-2, rashes, fevers, gastrointestinal involvement, and elevated inflammatory markers. The first two cases had multiple pleural effusions and required positive pressure ventilation and inotropes due to distributive shock. Interestingly, both cases did not have any coronary artery involvement or myocardial depression, diverging from previously reported cases of MIS-C [5]. The second case had a comparatively quicker recovery and shorter duration of inotropic support and positive pressure ventilation. The third case was milder in comparison without signs of shock or need for PICU admission.

The first case poses a diagnostic dilemma. While this patient has many features of MIS-C, his nasal swab was positive for MSSA, and thus toxic shock syndrome (TSS) was considered. The clinical criteria for TSS include the presence of streptococcal or staphylococcal infection along with fever, rash, hypotension, and multisystem organ involvement, often with bandemia, and leukocytosis or leukopenia. The CDC considers TSS probable if the criteria are met in the absence of another etiology [7]. Because the TSS case definition is so similar to that of MIS-C, there is little to distinguish between the two diagnoses in a patient who has both a bacterial source and recent SARS-CoV-2 exposure or infection with COVID-19. For this reason, particularly in the early stages of illness, it is important to manage patients with shock from suspected MIS-C with not only fluid resuscitation and vasoactive medications but also with broad-spectrum antibiotics until cultures are negative. 

While the second case falls into the case definition of MIS-C, he also met the criteria for incomplete Kawasaki disease. Incomplete Kawasaki disease identifies patients who have not yet had a fever for the required five days and have only two of the typical five principal clinical features of Kawasaki disease (oral erythema or cracking lips; bulbar conjunctival injection; maculopapular erythroderma; erythema, edema, and desquamation of the hands and feet; and unilateral cervical lymphadenopathy greater than 1.5 cm). Children with KD are typically less than five years old with a median age of two years of age, consistent with our patient’s age [8]. The etiology of KD has been debated in the literature, with no one causative agent consistently found; however, a superantigen-mediated immune activation has been proposed and may be similarly related to MIS-C [9]. TSS is also the result of superantigen and toxin activation of the immune system [10]. As both TSS and KD have similar features to MIS-C, SARS-CoV-2 may be inducing a similar activation of the immune system.

Although the third case was positive for SARS-CoV-2, her comparatively mild elevation of inflammatory markers and stable clinical course led the treatment team to be more suspicious of IgA vasculitis versus hand, foot, and mouth disease. Her skin exam illustrates the importance of correctly characterizing rashes as maculopapular, vesicular, erythematous, or petechial/purpuric. The erythematous rash in KD and TSS is a diffuse red skin caused by capillary congestion. Comparatively, the petechial/purpuric rash of IgA vasculitis is palpable, non-blanching, small red lesions caused by capillaries leaking blood into the skin. The rash from hand, foot, and mouth disease is characterized by vesicles due to involvement of the dermal-epidermal junction, leading to the formation of fluid-filled lesions [11]. There exists a difficulty in determining if a rash is characteristic of MIS-C, as rashes of varying description have been reported [12]. The first cases out of the UK were described to have “varying rashes”, and the Centers for Disease Control and Prevention (CDC) and American Academy of Pediatrics (AAP) guidance does not specify the type of rash [13,14]. The American College of Rheumatology describes the rash seen in MIS-C as “polymorphic, maculopapular, or petechial, but not vesicular" [15]. The variability in skin rash in patients with MIS-C contributes to the challenge of correctly diagnosing and managing pediatric patients with fever and rash in the ED.

## Conclusions

The emergence of MIS-C adds to the vast differential for a pediatric patient presenting to the ED with a fever and rash. The overlap between clinical features of KD, TSS, and MIS-C and varying skin findings may pose a diagnostic dilemma for health care providers and complicate management decisions. With the continuing SARS-CoV-2 pandemic, ED providers will need to be alert and have high suspicion when evaluating a child with a fever and a rash to properly identify children presenting with this serious illness. 
